# Subcutaneous Levodopa in Parkinson's Disease: A Systematic Review and Meta‐Analysis

**DOI:** 10.1111/ene.70506

**Published:** 2026-01-29

**Authors:** Matthew Burton, Duncan Marsden, Dhruv Harish, Peter Morris, Carl Heneghan, Ailsa Butler, David Nunan

**Affiliations:** ^1^ University of Oxford Medical Sciences Division Oxford UK; ^2^ Centre for Evidence‐Based Medicine University of Oxford Oxford UK

**Keywords:** clinical trials, levodopa, Parkinson disease, subcutaneous infusions, systematic review

## Abstract

**Background:**

Many Parkinson's disease patients receiving oral levodopa/carbidopa experience a troublesome wearing off effect. Higher doses to mitigate OFF‐time are limited by adverse effects occurring at peak dopamine levels, particularly dyskinesia. A novel strategy to reduce OFF‐time without increasing peak dopamine levels is the continuous subcutaneous infusion of levodopa/carbidopa, or their prodrug equivalents foslevodopa/foscarbidopa.

**Objectives:**

Assess whether subcutaneous infusion therapies safely reduce OFF‐time and improve quality of life scores compared to oral levodopa/carbidopa.

**Methods:**

We searched MEDLINE, Embase, CENTRAL and ICTRP up to 28th October 2024 for clinical trials comparing subcutaneous infusions of levodopa or foslevodopa to oral levodopa in Parkinson's disease.

**Results:**

Screening of 1114 records identified seven studies in which 725 patients received subcutaneous infusion regimens of levodopa/carbidopa (ND0612) (407 patients) or foslevodopa/foscarbidopa (318 patients). Moderate quality evidence indicated subcutaneous infusion reduced the daily duration of OFF‐time by 1.98 h (*p* = 0.0004). Moderate quality evidence indicated improvements in health‐related quality of life score PDQ‐39 (*p* = 0.0003) and sleep score PDSS‐2 (*p* = 0.02), but an increase in the rate of treatment‐emergent adverse events, mostly related to the infusion site (*p* = 0.04).

**Conclusions:**

Subcutaneous infusion therapies produce a clinically and statistically significant reduction in the duration of OFF‐time experienced by patients with Parkinson's disease, compared to oral levodopa/carbidopa. Patient experience is improved by a statistically, but not clinically, significant degree. There are increased adverse events, mostly related to the infusion site. Overall, subcutaneous infusion regimens could provide a meaningful alternative for Parkinson's disease patients who experience severe motor fluctuations with existing levodopa formulations.

## 1 Introduction

Current Parkinson's disease (PD) therapy aims to manage symptoms, as no disease‐modifying treatments are available. Pharmacological therapies include levodopa (given with a decarboxylase inhibitor such as carbidopa or benserazide), dopamine receptor agonists, catechol‐O‐methyltransferase (COMT) inhibitors and monoamine oxidase‐B (MAO‐B) inhibitors, all of which increase dopaminergic signalling [1]. While these treatments offer symptomatic relief, their use is accompanied by a range of side effects including dyskinesias, impulsive behaviour, hallucinations and motor fluctuations [2]. Complementary non‐pharmacological therapy such as deep brain stimulation, physiotherapy and speech therapy can contribute to holistic amelioration of PD symptoms [3].

The mainstay and prevailing first‐line PD therapy is oral levodopa, a dopamine precursor converted to dopamine in the striatum [4]. It is given in combination with a peripheral DOPA‐decarboxylase inhibitor such as carbidopa to minimise peripheral breakdown of levodopa. A major limitation to the efficacy of oral levodopa is the development of motor fluctuations, thought to be a result of intermittent delivery. This entails a combination of wearing off when dopamine levels are low, known as ‘OFF‐time’, and dyskinesia when dopamine levels are high. During ‘OFF‐time’, when levodopa fails to control Parkinsonian symptoms, patients may experience exacerbated motor symptoms such as tremor, gait freezing and rigidity and neuropsychiatric fluctuations, significantly impairing quality of life. Prevalence of OFF‐time is estimated to be 40% after 4–6 years of therapy but can appear as early as 5 months [5, 6]. Dyskinesia, manifesting as involuntary movement typically when dopamine levels peak, is the most frequent adverse effect of levodopa, developing in around 80% of PD patients [7]. The burden of dyskinesia and OFF‐time increases with disease progression and duration of levodopa treatment, reducing ON time without dyskinesia when parkinsonian symptoms are effectively managed.

Attempts to reduce OFF‐time by increasing the oral levodopa dose are limited since this also leads to increased dyskinesia and other adverse effects. These effects mirror the plasma concentration of levodopa, which fluctuates with its short half‐life. Steady plasma levels, achieved by constant intravenous infusion of levodopa, have been shown to eliminate motor fluctuations [8]. Levodopa/carbidopa intestinal gel (LCIG), as well as levodopa/entacapone/carbidopa intestinal gel (LECIG), are infusion strategies currently in clinical use to stabilise plasma levels by targeting a continuous levodopa infusion. Large‐scale clinical data has shown LCIG to reduce OFF‐time, dyskinesia duration and severity of PD symptoms. Subcutaneous infusions of levodopa or foslevodopa are an emerging alternative to avoid the need for surgical placement and management of a percutaneous jejunostomy tube [9]. Continuous subcutaneous infusion by wearable pumps is a system familiar to users of subcutaneous apomorphine, an adjunct currently available for use in PD treatment. Whilst the poor solubility of existing levodopa formulations has precluded a subcutaneous approach in the past, multiple formulations have been developed that are shown to achieve stable plasma concentrations [10].

Three formulations exist for subcutaneous levodopa delivery. ABBV‐951, known as foslevodopa/foscarbidopa, is a phosphate prodrug with high solubility to allow delivery of full daily doses with a small volume infusion [11]. ND0612 is a liquid formulation of levodopa/carbidopa, which differs in that it requires dual infusion sites and supplementary oral levodopa/carbidopa to meet daily dose requirements above 720 mg [12]. We deem it appropriate to analyse these formulations together (albeit separated in subgroup analysis) as they act identically once in the bloodstream, can reach similar peak plasma concentrations and produce similar fluctuation indices, and the latter two pharmacokinetic properties we deem most likely to affect OFF‐time. A third formulation, DIZ102, is a concentrated acidic levodopa/carbidopa solution designed to provide a monotherapy, also requiring two infusion sites [13]. No studies of DIZ102 meet the inclusion criteria of this review, as there are no published trials comparing it to oral levodopa/carbidopa.

This systematic review aims to be the first meta‐analysis to examine whether subcutaneous infusion of levodopa or foslevodopa can reduce daily OFF‐time as compared to oral levodopa in patients with Parkinson's disease. Further, this review aims to assess effects on quality of life scores, safety, and provide the first exploratory analyses of differences between the two formulations.

## 2 Methods

### 2.1 Source of Data and Search Strategy

We conducted our review in accordance with PRISMA guidelines and with guidance from the Cochrane Handbook for Systematic Reviews of Interventions [14, 15]. We registered the protocol on Open Science Framework: https://doi.org/10.17605/OSF.IO/MT652.

We searched the following databases for clinical trials: Ovid MEDLINE from 01/01/1946, Embase from 01/01/1974, Cochrane Central Register of Controlled Trials and International Clinical Trials Registry Platform, to Oct 28 2024. We formulated a comprehensive search strategy (Appendix S1), in collaboration with an information specialist, using a population, intervention, comparison, outcome (PICO) framework. Key search terms were ‘subcutaneous drug administration’ and ‘Parkinson*’.

### 2.2 Eligibility Criteria

We included randomised controlled trials and relevant clinical trials with non‐standard designs, such as pre‐post trials and open‐label trials. We included trials of any duration that compared subcutaneous infusions of any levodopa/carbidopa delivery formulation to oral levodopa/carbidopa. We included human trials performed in Parkinson's disease patients of any age or gender. Our primary outcome was reduction in OFF‐time experienced per day. Secondary outcomes included quality of life scales, adverse events and adherence. We included all studies with comparative data on at least one of our outcomes. We excluded studies not available in English due to a lack of resources for scientifically accurate translations.

### 2.3 Study Selection

After searching, two reviewers independently screened the abstracts of identified studies for eligibility. All relevant abstracts proceeded to full‐text screening by two reviewers. Any conflicts were first discussed by the two reviewers and, if consensus was not reached, by all four reviewers.

### 2.4 Data Extraction

We used a pre‐designed data extraction template. The data extracted included study setting, number of participants, population data, intervention and control details and relevant assessment time points. Data relating to outcomes included: OFF‐time per day, quality of life scales, adverse events and adherence rates. Information regarding funding and declarations of interest was also collected. Where data was missing, we requested it from the authors.

### 2.5 Data Synthesis

Where appropriate, we carried out meta‐analyses using RevMan software [16]. Where there was insufficient data for meta‐analysis, we compiled tables and summarised the findings narratively. Adverse effects reported by each study were compiled into table form.

We used a random effects model for meta‐analysis to mitigate the effects of heterogeneity in the study cohorts and methods. We calculated mean difference in our meta‐analyses of OFF‐time, PDSS‐2 and PDQ‐39 and risk ratios in our meta‐analyses of TEAEs and STEAEs. In all cases we calculated a 95% confidence interval and a two‐tailed *p*‐value and assessed heterogeneity using the Thompson *I* ^2^ statistic [14]. We anticipated a low number of TEAEs and STEAEs, so we used the Mantel–Haenszel method in our meta‐analyses.

For all outcomes, we conducted subgroup analysis separating trials using ND0612 from those using foslevodopa/foscarbidopa. We also conducted two sensitivity analyses on our primary outcome, the duration of OFF‐time per day. The first excluded long‐term trials > 26 weeks in duration. The second excluded trials lacking an independent control group. We did not use funnel plots to assess publication bias as there were fewer than ten studies included in our analysis.

We used the Cochrane GRADE framework [15] to estimate the overall certainty of evidence for our main findings.

### 2.6 Risk of Bias Assessments

We conducted risk of bias assessments for all included studies for all outcomes. This was performed independently by two reviewers. Any conflicts were first discussed by the two reviewers and, if consensus was not reached, by all four reviewers. We used Cochrane Risk of Bias 2 for randomised and crossover trials, and Risk of Bias in Non‐Randomised Studies—of Interventions (ROBINS‐I) tools as appropriate [17, 18].

### 2.7 Data Sharing and Accessibility

Research data are not shared in a public data repository.

## 3 Results

### 3.1 Description of Studies

#### 3.1.1 Results of the Search

**FIGURE 1:**
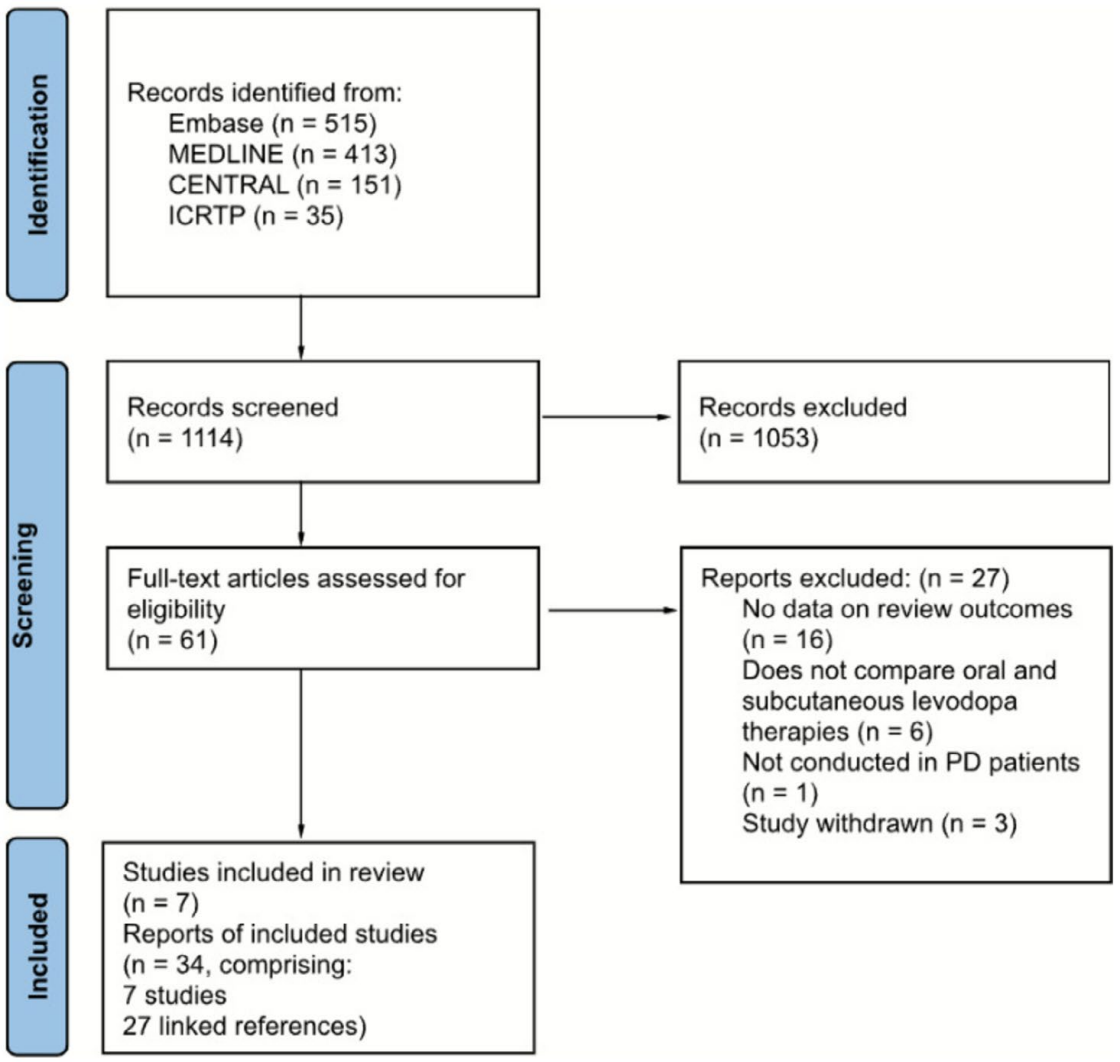
PRISMA flow diagram illustrating the results of the study selection process.

We screened 1114 records identified by our searches, assessed the full text of 61 articles for eligibility and included 34 eligible records from seven studies. Figure 1 shows the PRISMA study selection flow diagram.

#### 3.1.2 Included Studies

Table 1 shows nine intervention groups from seven included trials published between 2021 and 2024. We included records from three randomised controlled trials [20, 21, 22, 23, 24, 25, 26, 27, 34, 35], one cross‐over trial [12], two dosing‐regimen comparisons without independent control groups [19, 29, 30, 31, 32] and one single arm open‐label study [33]. Both dosing‐regimen comparison studies included two intervention groups which were analysed separately. Five trials were sponsored by NeuroDerm and two trials were sponsored by AbbVie. Studies were conducted across multiple centres in Europe, North America, Australia and Israel.

**TABLE 1:** Characteristics of included studies.

Study ID	Study design	Control type	Total number of participants	Mean participant age, years (SD)	Percentage female	Mean time since PD diagnosis, years (SD)	Daily infusion duration, hours	Treatment duration	Sole therapy/supplemented/adjunct	Mean total levodopa dose prior to study, mg (SD) [range‐used where SD not given]	Levodopa intervention dose, mg	Study start month	Year of primary results publication	Sponsor	Location	Setting
Espay et al. [19]	RCT	Oral Levodopa with dummy infusion	259	63.5 (9.0)	36.30%	9.3 (3.8)	24 h	12 weeks	Supplemented	1237 (447)	≤ 720	Aug‐19	2024	Neuroderm	Multicentre (Europe, Israel, USA, Russia)	Academic and community neurology sites
Soileau et al. [20, 21, 22, 23, 24, 25, 26]	RCT	Oral Levodopa with dummy infusion	141	66.4 (9.5)	30%	8.58 (4.85)	24 h	12 weeks	Sole therapy	1050 [800–1500]	NR	Jul‐20	2022	Abbvie	Multicentre (USA and Australia)	Academic and community study centres
Giladi et al. [12, 16, 27]	RCT	Oral Levodopa with dummy infusion	30	64.1 (7.1)	30%	8.6 (4.5)	24 h (16 h high rate, 8 h reduced rate)	2 weeks	Adjunct	660 (428)	270	Dec‐13	2021	Neuroderm	Multicentre (Israel)	Parkinson's medical centres
LeWitt et al. [27] (ND0612‐002)	Crossover	Oral Levodopa with dummy infusion	8	66.9 (5.3)	50%	NR	24 h (16 h high rate, 8 h reduced rate)	24 h	Adjunct	NR	270	Feb‐13	2022	Neuroderm	NR	NR
Aldred et al. [28]	Baseline controlled	N/A	244	63.9 (9.2)	40%	10.7 (5.2)	24 h	52 weeks	Sole therapy	1065 (585)	1860, 717	Apr‐2019 (estimated)	2023	Abbvie	Multicentre (Europe, USA, Japan, Australia)	NR
Poewe et al. [29] (24 h/day)	Baseline controlled	N/A	90	64.2 (8.9)	34%	9 (4.7)	24 h	52 weeks	Supplemented	1090 (623)	≤ 720	May‐16	2021	Neuroderm	Multicentre (Europe, Israel and USA)	NR
Poewe et al. [29] (16 h/day)	Baseline controlled	N/A	124	63.9 (8.9)	34%	9 (4.7)	16 h	52 weeks	Supplemented	1004 (540)	≤ 720	May‐16	2021	Neuroderm	Multicentre (Europe, Israel and USA)	NR
Olanow et al. [30, 31, 32, 33] (24 h/day)	Baseline controlled	N/A	19	63 (10.1)	33%	11.5 (5.2)	24 h	4 weeks	Supplemented	996 (552)	≤ 720	Dec‐15	2021	Neuroderm	Multicentre (Europe, Israel and USA)	Specialist PD sites
Olanow et al. [30, 31, 32, 33] (14 h/day)	Baseline controlled	N/A	19	64 (8.5)	32.60%	11.5 (5.2)	14 h	4 weeks	Supplemented	948 (305)	≤ 720	Dec‐15	2021	Neuroderm	Multicentre (Europe, Israel and USA)	Specialist PD sites

Abbreviation: NR, not reported.

All intervention groups received subcutaneous infusion of either ND0612 or foslevodopa/foscarbidopa. Intervention groups receiving foslevodopa received no oral levodopa (shown as ‘Sole therapy’ in Table 1) whilst intervention groups receiving ND0612 received supplementary oral levodopa where daily levodopa requirements exceeded the maximum subcutaneous dose deliverable (shown as ‘Supplemented’ in Table 1). Two intervention groups receiving ND0612 received only 270 mg of subcutaneous levodopa per day, with the majority of their daily levodopa being delivered orally (shown as ‘Adjunct’ in Table 1). Interventions differed in their daily duration of infusion, with foslevodopa generally administered over 24 h, and ND0612 administered either over 14–16 h or 24 h. Some studies with 24 h/day infusion duration employed a reduced overnight infusion rate. Total duration of treatment varied from 24 h to 52 weeks. In all studies apart from Olanow et al., OFF‐time was measured subjectively through a patient‐reported home diary. In Olanow et al., OFF‐time was measured by a blinded clinician over 8 h in a clinic setting.

### 3.2 Risk of Bias

Risk of bias assessments for all outcomes are presented in Figures S1–S5 and those relating to primary outcomes are discussed below.

#### 3.2.1 Randomised Controlled Trials

Espay et al. [27] and Soileau et al. [20, 21, 22, 23, 24, 35] were judged to be at low risk of bias. Giladi et al. [25, 26, 34] were judged to be of some concern, arising from missing detail on the randomisation process. Risk of bias judgements are displayed in Figure S1.

#### 3.2.2 Non‐Randomised Baseline‐Controlled Studies

Aldred et al. [33] and Olanow et al. [29, 30, 31, 32] were judged to be at serious risk of bias, due to lack of blinding. OFF‐time was measured through a patient‐reported PD diary in Aldred et al., meaning lack of blinding is unlikely to have introduced bias at the level of the investigators but may have impacted participants' perceptions of OFF‐time. In Olanow, OFF‐time was measured by blinded clinicians, minimising the effect of the study being otherwise unblinded. Aldred et al. [33] was judged to be at moderate risk of bias from confounding since treatment efficacy was compared at 52 weeks versus baseline, and during this time it is likely some patients had a progression of their Parkinson's disease. We note that this bias would be likely to understate, rather than overstate, the true benefit of treatment. Olanow et al. [29, 30, 31, 32] was judged to be less at risk of this confounding since treatment efficacy was compared at 4 weeks versus baseline, leaving less opportunity for meaningful PD symptom progression. Overall and domain specific risk of bias judgements are displayed in Figure S2.

#### 3.2.3 Other Potential Sources of Bias

All studies were funded by, and had authors affiliated with, the pharmaceutical company producing the product used. This should be taken into account when considering the risk of bias in all outcomes of these studies.

### 3.3 Synthesis

#### 3.3.1 OFF‐Time Per Day

We conducted a meta‐analysis investigating the duration of OFF‐time experienced per day by patients receiving subcutaneous infusions of ND0612 or foslevodopa/foscarbidopa compared to those receiving oral levodopa. This meta‐analysis included data from Soileau et al. [20, 21, 22, 23, 24, 35], Olanow et al. [29, 30, 31, 32], Giladi et al. [25, 26, 34], Espay et al. [27] and Aldred et al. [33]. Two studies were excluded: LeWitt et al. [12] contained no original data on OFF‐time; Poewe et al. [19] collected data on OFF‐time but did not present it in sufficient detail. In all studies apart from Olanow et al., OFF‐time was assessed through the use of a patient‐reported home diary. In Olanow et al., OFF‐time was measured by a blinded clinician over 8 h in a clinic setting. The data were combined to produce a mean difference of the change in OFF‐time experienced per day, in hours. The included data are presented in 2A, with subgroup analysis separating studies which investigated ND0612 from those which investigated foslevodopa/foscarbidopa.

**FIGURE 2:**
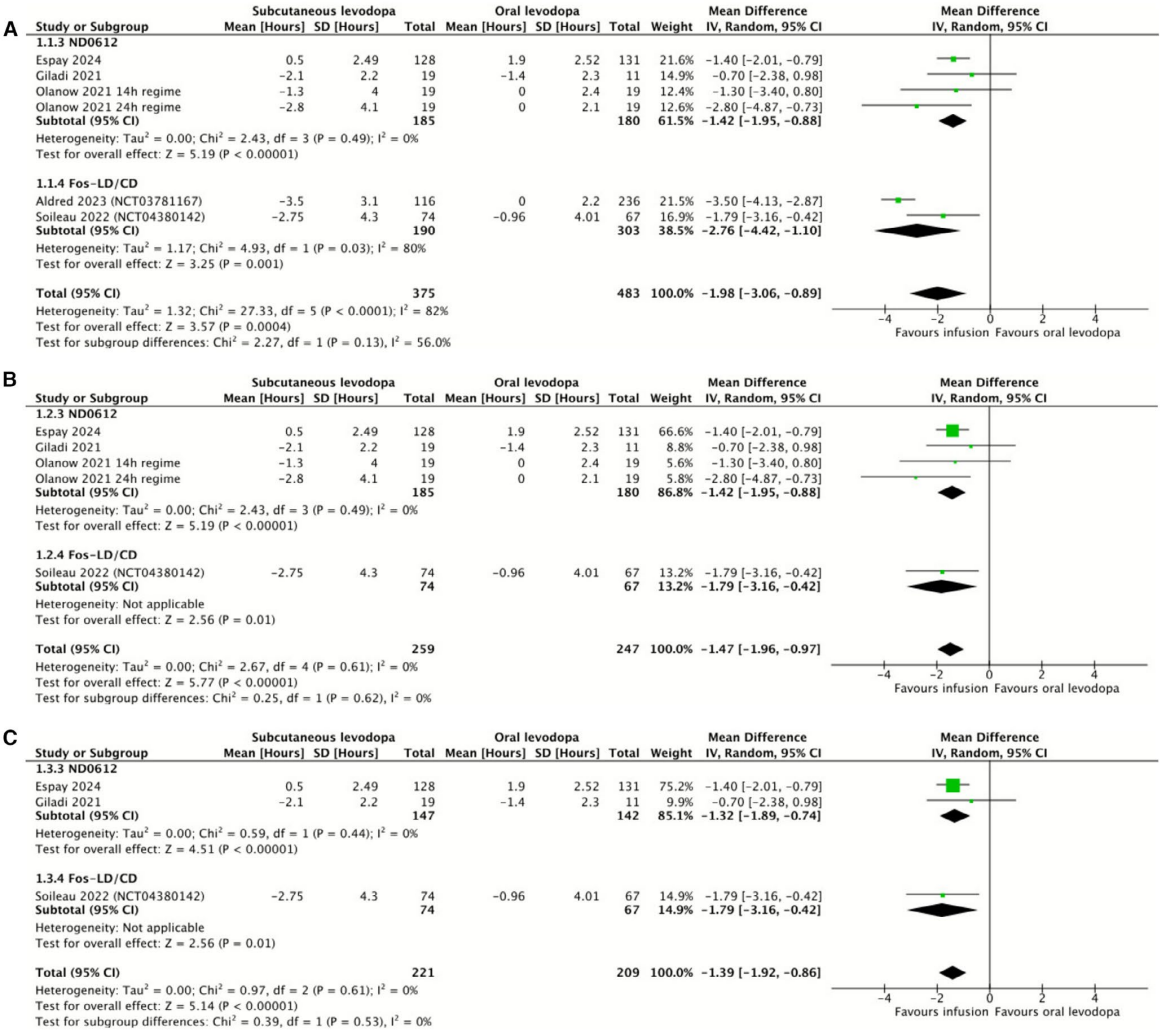
Forest plots comparing change in OFF‐time per day (hours) between subcutaneous infusion regimens and oral levodopa/carbidopa. Subgroup analysis separates studies with the subcutaneous levodopa/carbidopa formulation ND0612 from those with the foslevodopa/foscarbidopa formulation. (A) Change in OFF‐time per day (hours) between subcutaneous infusion regimens and oral levodopa/carbidopa. (B) Sensitivity analysis excluding studies greater than 26 weeks in duration. (C) Sensitivity analysis excluding studies without an independent control group.

Our meta‐analysis showed that subcutaneous infusion therapies achieved a mean reduction of 1.98 h in the duration of OFF‐time experienced per day compared to oral levodopa/carbidopa. This represented a statistically significant difference (Figure 2A). For ND0612 alone, this reduction was 1.42 h per day, and for foslevodopa/foscarbidopa alone, this reduction was 2.76 h per day, both statistically significant. In the overall analysis, we detected considerable heterogeneity of 82% using the Thompson I^2^ statistic. When we performed subgroup analysis, the heterogeneity in the ND0612 studies was measured to be 0%, whereas in the foslevodopa studies, the heterogeneity was measured as 80%. We proceeded to investigate this further.

We performed sensitivity analysis to identify the impact of study duration, excluding Aldred et al., which followed patients for 52 weeks, whereas the next longest studies followed patients for 12 weeks (Figure 2B). The overall reduction in OFF‐time compared to oral levodopa was reduced to 1.47 h but remained statistically significant. For ND0612 the benefit was 1.42 h and for foslevodopa/foscarbidopa 1.79 h, both remaining statistically significant. Heterogeneity in the overall analysis was reduced to 0%, with no statistically significant difference between subgroups. We believe this likely reflects the impact of the long follow‐up duration in Aldred et al., although as only two foslevodopa/foscarbidopa studies were included in our original meta‐analysis it is difficult to rule out other sources of heterogeneity between those two studies.

We conducted a sensitivity analysis including only studies with independent control groups (Figure 2C). This meant that all studies with a serious risk of bias were excluded. In this analysis, the overall reduction in OFF‐time compared to oral levodopa was lower, at 1.39 h, but was still statistically significant. For ND0612, the benefit was 1.32 h, whereas for foslevodopa/foscarbidopa the benefit remained 1.79 h, both statistically significant. Heterogeneity as detected by the Thompson I^2^ statistic was 0% in all analyses. This shows that the benefit found in our primary analysis is robust to the exclusion of studies without independent controls and that are therefore at a serious risk of bias.

#### 3.3.2 Quality of Life Scores

**FIGURE 3:**
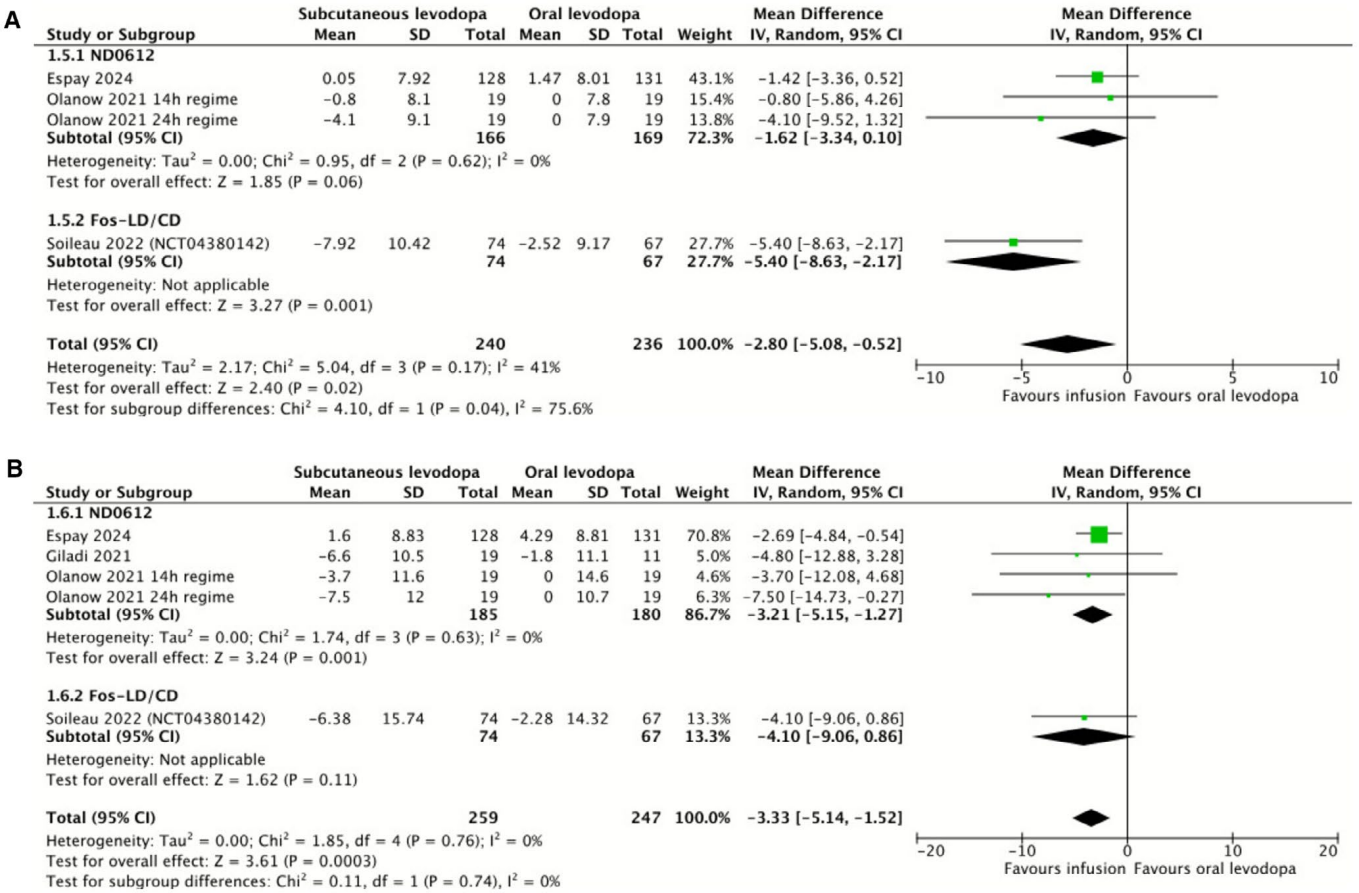
Forest plot comparing changes in patient disease‐rating scales compared to baseline between subcutaneous infusion regimens and oral levodopa/carbidopa. Subgroup analysis separates studies with the subcutaneous levodopa/carbidopa formulation ND0612 from those with the foslevodopa/foscarbidopa formulation. (A) Change in PDSS‐2 scores compared to baseline. PDSS‐2, Parkinson's Disease Sleep Scale‐2. (B) Change in PDQ‐39 scores compared to baseline. PDQ‐39, Parkinson's Disease Questionnaire‐39.

We conducted meta‐analyses on the patient scoring systems PDSS‐2 (for sleep symptoms) and PDQ‐39 (a comprehensive health‐related quality of life score), as shown in Figure 3. All studies where data was presented in sufficient detail were included. We also extracted data on the UPDRS/MDS‐UPDRS and EQ‐5D‐5L scales. Three studies reported UPDRS data (only one reporting all parts of UPDRS), two studies reported MDS‐UPDRS data and two studies reported EQ‐5D‐5L data. We judged that this data was insufficient for a meta‐analysis to be appropriate. All available data was presented in Table S1.

Meta‐analysis of PDSS‐2 data found a statistically significant improvement in scores with subcutaneous infusion therapy (Figure 3A). However, there was moderate heterogeneity and a statistically significant difference between subgroups. ND0612, analysed alone, was associated with an improvement of 1.62 in PDSS‐2 score, with no statistical significance but no heterogeneity. Soileau et al., the sole foslevodopa/foscarbidopa study which could be included, was associated with a statistically significant improvement of 5.4 in PDSS‐2 score. Meta‐analysis of PDQ‐39 data found a statistically significant improvement associated with subcutaneous infusion therapy compared to oral levodopa (Figure 3B). Heterogeneity was 0%. We note that Soileau et al., the sole foslevodopa study with sufficient PDQ‐39 reporting, showed a similar effect size to other studies but markedly increased standard deviation, so did not show a statistically significant improvement when analysed alone.

#### 3.3.3 Adverse Events

**FIGURE 4:**
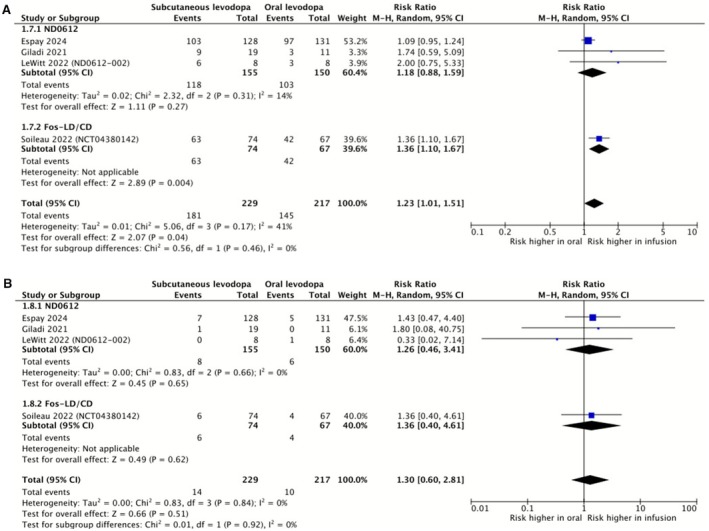
Forest plots comparing incidence of (A) Treatment‐emergent adverse events (TEAEs) and (B) Serious treatment‐emergent adverse events (STEAEs) between subcutaneous infusion regimens and oral levodopa/carbidopa. Subgroup analysis separates studies with the subcutaneous levodopa/carbidopa formulation ND0612 from those with the foslevodopa/foscarbidopa formulation.

We conducted meta‐analyses on the incidence of treatment‐emergent adverse events (TEAEs) and serious treatment‐emergent adverse events (STEAEs) with subcutaneous infusions of ND0612 or foslevodopa/foscarbidopa compared to oral levodopa (Figure 4). Data from Espay et al. [27], Giladi et al. [25, 26, 34], LeWitt et al. [12] and Soileau et al. [20, 21, 22, 23, 24, 35] were included. Aldred et al. [33], Poewe et al. [19] and Olanow et al. [29, 30, 31, 32] could not be included as they lacked a control group for comparison. The Mantel–Haenszel method was used due to the small number of data points.

When the incidence of TEAEs and STEAEs was analysed across studies, the relative risk appeared to be increased by the subcutaneous infusions compared to oral levodopa; however, this was only statistically significant for TEAEs (Figure 4A), not for serious TEAEs (Figure 4B). In the case of TEAEs, moderate heterogeneity was detected using the Thompson *I* ^2^ statistic, whereas low levels of heterogeneity were detected in the case of STEAEs. Comparing ND0612 to foslevodopa/foscarbidopa, there was no statistically significant difference between subgroups. Across all studies, the majority of TEAEs reported in patients receiving subcutaneous infusions related to the infusion site, including erythema, pain, oedema, bruising, nodule formation, infection and pruritus. The incidence of systemic side effects appears to be in line with expectations for Parkinson's disease patients receiving dopamine replacement therapy. Reported TEAEs meeting each study's respective reporting criteria are collated in Table S2. Looking to the large, 52‐week, single‐arm studies, Aldred et al. and Poewe et al., systemic adverse events affecting over 10% of participants in either study were hallucination, fall, anxiety, dizziness, nausea and urinary tract infection. Looking to the two large RCTs, Soileau et al. and Espay et al., for comparative data, hallucination met reporting criteria in Soileau, occurring in 1% and 7% (1 and 5 participants) of the control and study drug groups, respectively. 1 participant (1%) in the foslevodopa/foscarbidopa study drug group discontinued study drug treatment due to hallucination or psychosis. In Espay et al., hallucination did not meet reporting criteria. This may be in part owing to an exclusion criterion of acute psychosis or troublesome hallucinations in the previous 6 months. Incidence of falls was lower in study drug groups compared to control groups in both RCTs. Anxiety met reporting criteria in Espay et al. and had a lower incidence in the study drug group compared to the control group. Dizziness was not reported in either trial; however, the incidence of balance disorder met reporting criteria in Soileau with an incidence of 5% (4 participants). Neither nausea or urinary tract infection were reported in Esapy et al. or Soileau et al. In summary, hallucination is the only frequently reported adverse effect that appears to have an increased incidence with subcutaneous delivery of levodopa. Dyskinesia is of particular interest as it is the adverse treatment effect of levodopa which typically limits oral dosing. In the two large RCTs, there was a greater incidence of dyskinesia with foslevodopa/foscarbidopa compared to oral levodopa/carbidopa in Soileau et al., with an incidence of 11% and 6% (8 and 4 participants), respectively. In Espay et al., there was a lower incidence of dyskinesia with ND0612 compared to oral levodopa/carbidopa, with an incidence of 2% and 4% (3 and 5 participants), respectively. Despite the greater incidence of dyskinesia as an adverse event with foslevodopa in Soileau et al. [23, 24, 35], the study drug tends towards a reduction in median PKG dyskinesia score, treatment difference −2.73 (95% CI −6.61 to 1.15).

#### 3.3.4 Adherence and Withdrawals

Data on adherence and withdrawals from each study are presented in Table S3. There is notable variation in the completion rates across different studies, with particularly low rates in the studies with 52‐week follow‐up (Aldred et al. [33] and Poewe et al. [19]). In both, significant numbers withdrew due to adverse events; however, both studies lacked control groups and so relative risks could not be calculated. We note that Aldred et al. [33] state 137 patients completed treatment, giving the completion rate of 56.1% presented in Table S3; however, data was only made available for 116 patients. This missing data has been considered in our risk of bias assessment.

### 3.4 Summary of Findings

We have presented a summary of our findings in Table 2. Where the certainty of evidence for a finding has been downgraded, the explanation for this is presented below the table, alongside an explanation of the GRADE levels of certainty of evidence.

**TABLE 2:** Summary of findings table.

Outcome		Mean difference between subcutaneous and oral therapy (95% CI)	Relative risk of TEAEs (95% CI)	Number of participants (studies)	Certainty of evidence (GRADE)	Key message
Change in OFF‐time per day (hours)	All results	−1.98 (−3.06, −0.89)		483 (5)	Moderate^a^	Subcutaneous infusion regimens are likely to cause a reduction in OFF‐time per day compared to oral levodopa/carbidopa in PD patients, in the range of 0.89–3.06 h per day.
	Studies < 26w in duration only	−1.47 (−1.96, −0.97)		247 (4)	Moderate^b^	When excluding studies beyond 26 weeks in duration, subcutaneous infusion regimens remain likely to cause a reduction in OFF‐time per day, in the range of 0.97–1.96 h per day.
	Studies with an independent control only	−1.39 (−1.92, −0.86)		209 (3)	High^c^	When excluding studies lacking independent controls, subcutaneous infusion regimens remain likely to cause a reduction in OFF‐time per day, in the range of 0.86–1.92 h per day.
PDSS‐2		−2.80 (−5.08, −0.52)		236 (3)	Moderate^d^	Subcutaneous infusion regimens are likely to cause an improvement in sleep quality as assessed by PDSS‐2 when compared to oral levodopa/carbidopa. The certainty of evidence for this finding is downgraded due to lack of blinding in one study for this relatively subjective measure.
PDQ‐39		−3.33 (−5.14, −1.52)		247 (4)	Moderate^e^	Subcutaneous infusion regimens are likely to cause an improvement in overall disease burden as assessed by PDQ‐39 when compared to oral levodopa/carbidopa.
TEAEs			1.23 (1.01, 1.51)	217 (4)	Moderate^f^	Subcutaneous infusion regimens are likely to be associated with a modestly increased rate of adverse events compared to oral levodopa/carbidopa in PD patients.
STEAEs			1.30 (0.60, 2.81)	217 (4)	Low^g^	We did not find evidence to suggest that subcutaneous infusion regimens affect the rate of serious adverse events in PD patients.

*Note:* GRADE Working Group grades of evidence. High certainty: we are very confident that the true effect lies close to that of the estimate of the effect. Moderate certainty: we are moderately confident in the effect estimate: the true effect is likely to be close to the estimate of the effect, but there is a possibility that it is substantially different. Low certainty: our confidence in the effect estimate is limited: the true effect may be substantially different from the estimate of the effect. Very low certainty: we have very little confidence in the effect estimate: the true effect is likely to be substantially different from the estimate of the effect.

Abbreviations: PDQ‐39, Parkinson's Disease Questionnaire‐39; PDSS‐2, Parkinson's Disease Sleep Scale‐2; STEAs, serious treatment‐emergent adverse events; TEAEs, treatment‐emergent adverse events.

^a^ Downgraded once for study limitations: serious risk of bias in two studies.

^b^ Downgraded once for study limitations: serious risk of bias in one study.

^c^ Not downgraded for risk of bias, inconsistency, indirectness, imprecision, or publication bias.

^d^ Downgraded once for study limitations: serious risk of bias in one study.

^e^ Downgraded once for study limitations: serious risk of bias in one study.

^f^ Downgraded once for study limitations: imprecision of result.

^g^ Downgraded twice for inconsistency and imprecision.

## 4 Conclusion

### 4.1 Summary of Main Results

After identifying 1114 records in our search strategy, seven studies were deemed eligible for inclusion in our analysis, with 483 patients included in our primary outcome meta‐analysis.

We found moderate certainty evidence that treating Parkinson's disease patients with subcutaneous infusions of ND0612 or foslevodopa/foscarbidopa results in a large reduction in the duration of OFF‐time experienced by the patients per day, compared to oral levodopa/carbidopa [28]. This evidence is composed of both independently controlled trials and non‐randomised clinical trials. When we analysed only independently controlled trials, the magnitude of the benefit remained large (although somewhat reduced), and the evidence was found to be of high certainty. This provides strong evidence for the benefit of ND0612 and foslevodopa/foscarbidopa in reducing OFF‐time.

In our overall analysis of OFF‐time there was substantial Thompson *I* ^2^ heterogeneity of 82%. We hypothesised that the source of this heterogeneity might have been our inclusion of Aldred et al., a study with 52 weeks follow‐up, alongside the other studies which had a maximum of 12 weeks follow‐up. In a sensitivity analysis of study duration we excluded Aldred et al. and overall heterogeneity was reduced to 0%. This analysis continued to show a statistically significant reduction in daily OFF‐time with moderate certainty evidence. All studies, with either formulation, showed a similar benefit to daily OFF‐time once Aldred et al. was excluded.

We note that in the long‐term study Aldred et al., the benefit of foslecodopa/foscarbidopa was greater than any other study, but the withdrawal rate was also higher. We believe this may reflect self‐selection bias, with a subgroup of patients who respond best to infusion therapy opting to continue treatment whereas others withdraw. This clearly indicates subcutaneous infusions can provide a long‐term symptomatic benefit in at least a subgroup of patients.

We found moderate quality evidence for a statistically significant improvement in PDSS‐2 and PDQ‐39 quality of life scores in patients treated with subcutaneous ND0612 or foslevodopa/foscarbidopa. While this effect was consistent across subgroups on the PDQ‐39 scale, subgroup analysis on the PDSS‐2 scale revealed a statistically significant difference between ND0612 and foslevodopa/foscarbidopa, the latter being associated with a greater benefit. This may reflect that the pharmacodynamics of foslevodopa/foscarbidopa permit a greater plasma concentration of levodopa to be maintained overnight, reducing sleep symptoms. In general, our analysis of quality of life scales was limited by the variable selection and reporting of scales across studies, and we note this conclusion is limited as only Soileau et al. reported data on PDSS‐2 with foslevodopa/foscarbidopa.

Analysis of adverse events found evidence of moderate certainty that subcutaneous infusion of ND0612 or foslevodopa/foscarbidopa was associated with a 23% increased incidence of TEAEs, but no evidence to suggest an increased rate of STEAEs. We found that there was no statistically significant difference in the rates of TEAEs or STEAEs between the subgroup infused with ND0612 and the subgroup infused with foslevodopa/foscarbidopa. The majority of the adverse events were swelling, bruising, inflammation, infection or pain at the infusion site. Systematic reviews of levodopa/carbidopa intestinal gel (LCIG) report comparable rates of adverse events, with local infusion site reactions common [9]. In the two 52‐week studies we analysed, 23% (Aldred et al.) and 17.3% (Poewe et al.) discontinued due to adverse events.

Frequently reported systemic adverse events did not appear to have increased incidence in the treatment groups of the large RCTs when compared to the control groups, besides hallucination, which was responsible for one discontinuation. It is possible that continuous subcutaneous infusion of foslevodopa/Kumafoscarbidopa is associated with a greater incidence of hallucination than oral levodopa/carbidopa; this should be assessed with larger scale data.

Dyskinesia was reported to occur in > 10% of participants in one study of foslevodopa/foscarbidopa (Soileau et al. [23, 24, 35]); however, the same study found a non‐significant decrease in median wearable device dyskinesia score associated with foslevodopa treatment compared to control. Importantly, both of the larger RCTs included in this review found an increase in ‘on time without troublesome dyskinesia’. This suggests that the decrease we observe in OFF‐time does not come at the expense of increased time with troublesome dyskinesia.

### 4.2 Strengths and Limitations of the Review Process

This review was based on a protocol published in advance of the review. The protocol was written in accordance with PRISMA guidelines and with guidance from the Cochrane Handbook for Systematic Reviews of Interventions [14, 36].

We tried to identify all relevant studies through a comprehensive search strategy of publication databases and trial registries, designed in consultation with an information specialist, with no restrictions by date.

Screening and data extraction were performed independently and in duplicate. Disagreements were resolved by consensus discussion among the team.

All studies were assessed for risk of bias using the appropriate Cochrane tool, either RoB2.0 or ROBINS‐I, and this was also performed independently in duplicate [17, 18].

### 4.3 Overall Completeness and Applicability of Evidence

The patients included in this review followed a sex distribution typical of the wider population with Parkinson's disease [37]. Their ages and the time that had elapsed since their diagnosis were closely representative of the population believed to experience OFF‐time most commonly: younger patients who have had their diagnosis for a longer time [38].

The included studies were conducted in high‐resource settings. No conclusions can be drawn on the feasibility of the intervention in medium‐ and low‐resource settings.

In all studies apart from Olanow et al., OFF‐time was measured subjectively through a patient‐reported home diary. In Olanow et al., OFF‐time was measured by a blinded clinician over 8 h in a clinic setting. Home diaries have the benefit of reflecting a patient's own experiences of OFF‐time well but are of course subject to recall bias. Confirmatory data provided by Olanow et al. using blinded clinicians to score symptoms of OFF‐time are valuable in limiting this risk of bias. Although a range of disease rating and quality of life scales were used, some based on clinical assessment and others on questionnaires, the two most widely reported scales, PDQ‐39 and PDSS‐2, are both based on subjective questionnaires. Similarly, these methods produce results which are highly applicable to patients' own experiences of their disease and quality of life, but are also subject to recall bias.

We note that oral levodopa/carbidopa is not the only treatment currently available or undergoing research for use in Parkinson's disease. Due to a lack of studies comparing subcutaneous infusion therapies with novel therapies, we were restricted to comparing infusion therapies with the current oral standard of care. An important next step will be to compare subcutaneous infusion therapies with LCIG and LECIG.

### 4.4 Implications for Practice and Research

To contextualise our findings, we compared the observed reduction in OFF‐time with the mean daily OFF‐time at baseline. We found a mean reduction of 1.98 h, 34% of the mean daily OFF‐time of 5.81 h at baseline in the studies in this analysis (Figure 2A).

To establish clinical relevance, we looked to previous work to establish a minimum clinically important difference (MCID). A study investigating MCID for OFF‐time in trials of extended and immediate release pramipexole established values of 1.0 and 1.3 h respectively [39]. Our analysis found a mean reduction of 1.98 h, thus exceeding the MCID and suggesting clinical significance. Crucially, this remains the case when the analysis is limited to independently controlled trials, in which we found a mean reduction of 1.39 h. It was also retained whether studies had a shorter (< 26 weeks) or longer (52 weeks) follow‐up duration. We also note that these values represent a greater treatment difference than was found in a systematic review and meta‐analysis of LCIG [40].

We also found MCID values in the literature for PDSS‐2 [41] and PDQ‐39 [42] scores, of −3.44 and −4.72 respectively. Neither our finding of PDSS‐2 change of −2.80 nor our finding of PDQ‐39 change of −3.33 surpasses its MCID. Notably, in subgroup analysis foslevodopa/foscabridopa produced a clinically significant benefit of −5.40 on the PDSS‐2 scale; however, this reflects the result of a single RCT, Soileau et al. Our analysis of effect on quality‐of‐life scores was limited by the use of different scales between studies, with only a subset reporting PDQ‐39 and other scales not reported frequently enough to meta‐analyse. It appears that patient report of the benefits of subcutaneous ND0612 and foslevodopa/foscarbidopa may be limited, particularly in patient groups already established on oral treatment. Further evidence is needed to draw strong conclusions about the possible benefits of foslevodopa/foscarbidopa on sleep.

A qualitative report following up patients from Soileau et al. [20, 21, 22, 23, 24, 35] study, which continued with subcutaneous foslevodopa/foscarbidopa for 6 months, provides further insight into the impact of treatment‐emergent adverse events on patients [43]. Common issues included skin site events and problems using the device. These seemed to be troublesome particularly in the initial period, and the introduction of set routines and more healthcare support minimised the incidence of such events. All patients reported high levels of satisfaction and a high likelihood of continuing the therapy after the trial. It is possible that improved training of patients, carers and healthcare staff might have reduced the incidence of adverse events and withdrawals in some studies. As Parkinson's patients require lifelong dopamine therapy, trials with extended follow‐up periods may provide further insights into how patient experiences with this therapy can be optimised.

### 4.5 Agreements and Disagreements With Other Studies or Reviews

To date we know of no other meta‐analyses comparing ND0612 and foslevodopa/foscarbidopa with oral levodopa. We have aimed to provide a broad report on the key outcomes pertaining to this new therapy.

### 4.6 Conclusion

This systematic review finds that subcutaneous infusion with levodopa/carbidopa (ND0612) or foslevodopa/foscarbidopa produces a clinically and statistically significant reduction in the duration of OFF‐time experienced by patients with Parkinson's disease, compared to oral levodopa/carbidopa. This does not appear to be at the expense of increased dopamine‐related adverse events. We find that this is accompanied by improvements in patient experience that are statistically but not clinically significant. We find this treatment is linked with a modest increase in treatment‐emergent adverse events, which primarily relate to the injection site. Although not designed to allow rigorous comparison of foslevodopa/foscarbidopa against ND0612, our exploratory analyses indicate the two formulations have similar effects on OFF‐time, general patient experience and adverse events, with some limited evidence suggesting foslevodopa/foscarbidopa additionally reduces sleep symptoms. Further work, including in training patients, carers and healthcare professionals on the delivery of treatment could be valuable in optimising safety and tolerability.

## Author Contributions

**Matthew Burton:** Data Curation, formal analysis, methodology, writing – original draft preparation and review and editing. **Duncan Marsden:** Data curation, formal analysis, methodology, writing – original draft preparation and review and editing. **Peter Morris:** Data curation, formal analysis, methodology, writing – original draft preparation and review and editing. **Dhruv Harish:** Data curation, formal analysis, methodology, writing – original draft preparation and review and editing. **Carl Heneghan:** Supervision, writing – review and editing. **Ailsa Butler:** Supervision, writing – review and editing. **David Nunan:** Supervision, writing – review and editing.

## Funding

The authors have nothing to report.

## Ethics Statement

The authors conducted this review with the ethical guidelines laid out by the PRISMA 2020 statement in mind. Informed patient consent was not necessary for this work. We confirm that this work is consistent with the Journal's position on issues involved in ethical publication.

## Conflicts of Interest

The authors declare no conflicts of interest.

## Supporting information


**Data S1:** ene70506‐sup‐0001‐DataS1.docx.

## Data Availability

The authors have nothing to report.
