# Supplementary material for: Subcutaneous Levodopa in Parkinson's Disease: A Systematic Review and Meta‐Analysis
Source: Eur J Neurol. 2026 Jan 29;33(2):e70506. doi: 10.1111/ene.70506 (PMC12853972; doi:10.1111/ene.70506)
Supplement: Supplementary file 1 — Data S1: ene70506‐sup‐0001‐DataS1.docx. [file ENE-33-e70506-s001.docx]

**Supplementary materials**

**Figure S1: Rob2 assessment outcomes for randomised controlled trials in relation to OFF-time**

**
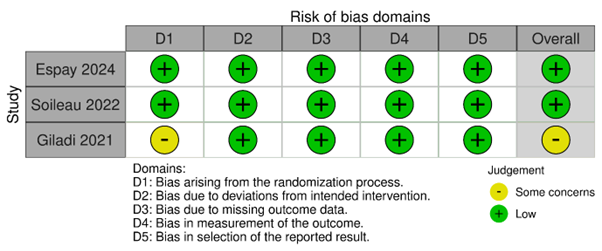
**

**Figure S2: Rob2 assessment outcomes for non-randomised trials in relation to OFF-time**

**
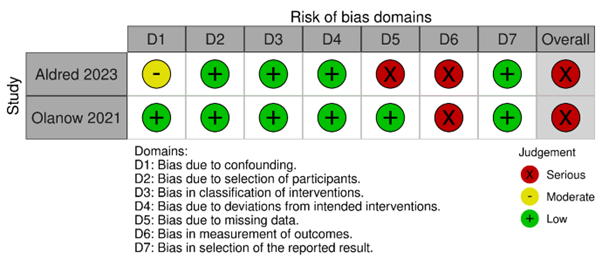
**

**Figure S3: Rob2 assessment outcomes for randomised-controlled trials reporting secondary outcomes**


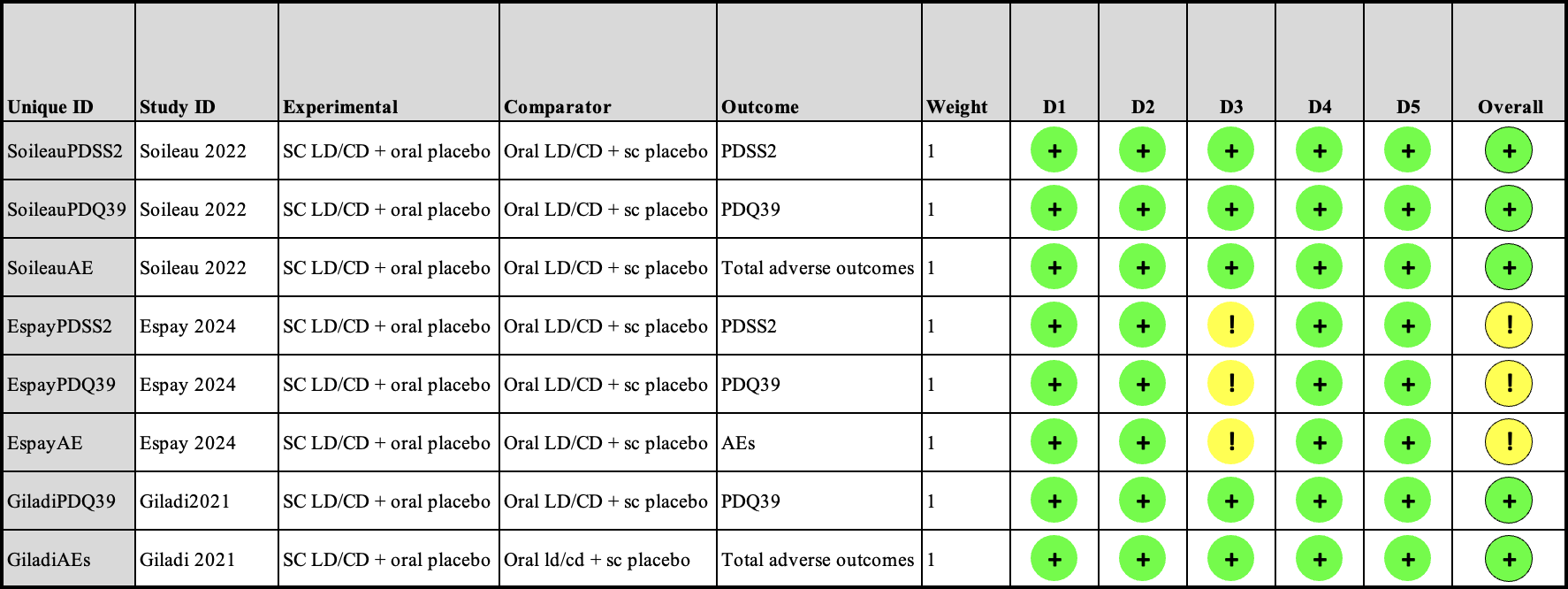


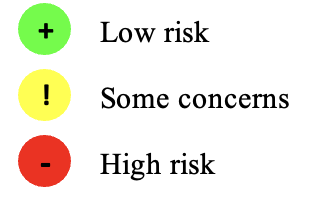

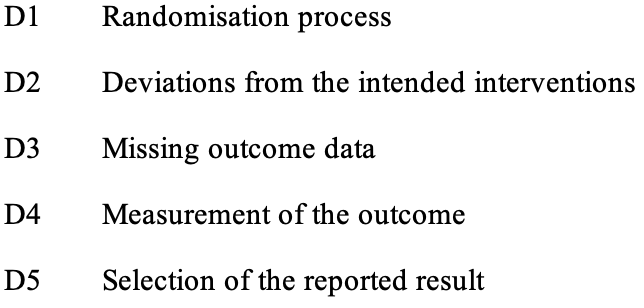


**Figure S4: RoB2 cross-over assessment of trial using randomised cross-over design for secondary outcomes**

**
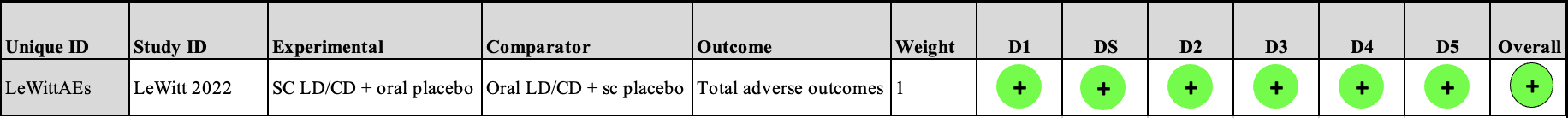
**

**
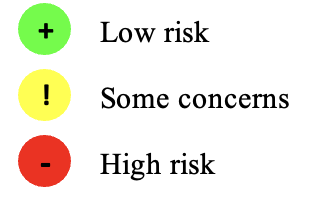

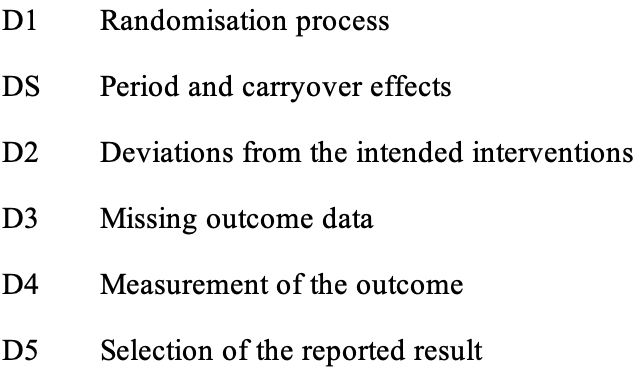
**

**Figure S5: ROBINS-I assessment of non-randomised trial for secondary outcomes**
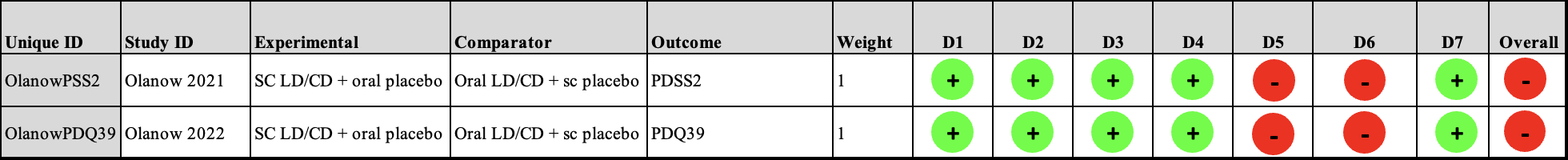


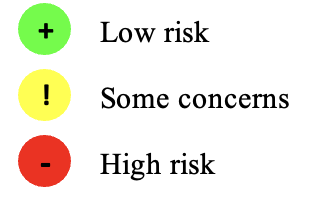

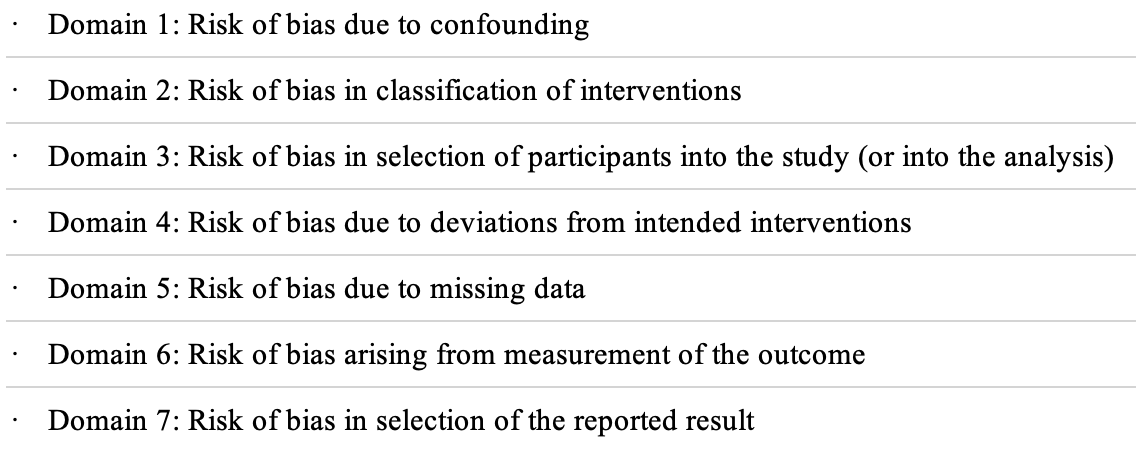


**Table S1: UPDRS or MDS-UPDRS and EQ-5D-5L score changes compared to baseline in subcutaneous and oral dopamine replacement regimens. NA= not assessed.**

| **Study** | **UPDRS**  **(treatment vs control ± SD) OR (change from baseline ±SD)** | | **MDS-UPDRS**  **Treatment difference (95% CI)** | **EQ-5D-5L**  **(treatment vs control ± SD) OR (change from baseline ±SD)** |
| --- | --- | --- | --- | --- |
| Aldred 2023 | NA | | Change from baseline not significant (data not provided). | Summary index:  0.097 ±0.2134 |
| Espay 2024 | NA | | Part II:  -3.05 (-4.28 to -1.81)  Part III:  -2.42 (-5.2 to 0.37) | NA |
| Giladi 2021 | UPDRS total:  -11.7 ±14.5 vs  -9.3 ±12.7 | Part IVa dyskinesia:  -0.7 ±1.4 vs  -0.1 ±1.0 | NA | Data not reported. |
| Olanow 2021 14h/day regime | Part II:  -1.9 ±5.1 | Part III:  -10.7 ±13.6 | NA | Data not reported. |
| Olanow 2021 24h/day regime | Part II:  -2.9 ±5.5 | Part III:  -19.1 ±14.6 | NA | Data not reported. |
| Soileau 2022 | NA | | Part II:  -1.58 (-3.65 to 0.48) | Summary index:  0.051 ±0.022 vs  0.002 ±0.021 |

**Table S2: Specific TEAEs reported to be frequent in each study. All figures represent number of patients (% of cohort)**

| **Study** | | | **Aldred et al (2023)^21^** | **Giladi et al (2021)^30-32^** | **LeWitt et al (2022)^29^** | **Olanow et al (2021) (14h/day)^34-37^** | **Olanow et al (2021) (24h/day)^34-37^** | **Poewe et al (2021) (16h/day)^33^** | **Poewe et al (2021) (24h/day)^33^** | **Soileau et al (2022)^22-28^** | **Espay et al (2024)^45^** |
| --- | --- | --- | --- | --- | --- | --- | --- | --- | --- | --- | --- |
| **Threshold for reporting** | | | Occurring in >10% of  patients | More than 2 patients  affected | NR | 2 or more patients affected (both study regimes) | 2 or more patients affected (both study regimes) | Occurring in >5% of  patients | Occurring in >5% of  patients | Occurring in >5% of  patients | Occurring in >5% of  patients |
| **Number  of TEAEs  (percentage  affected)** | **Infusion  site  related** | *Erythema* | 127 (52.0) | - | 12(9) | - | 5(26.3) | 7(5.6) | 9 (10.0) | 20 (27) | 12 (9) |
|  |  | *Nodule* | 70 (28.7) | - | - | - | 11(57.9) | 35(28.2) | 31 (34.4) | 6 (8) | 33 (26) |
|  |  | *Eschar* | - | - | - | - | - | 15(12.1) | 2 (2.2) | 4 (5) | - |
|  |  | *Infection* | 56 (23.0) | 13(10) | - | - | - | 11(8.9) | 15 (16.7) | 14 (19) | 13 (10) |
|  |  | *Oedema* | 47 (19.3) | - | - | 1(5.3) | 1(5.3) | 5(4) | 5 (5.6) | 9 (12) | - |
|  |  | *Pain* | 38 (15.6) | - | 11 (9) | - | 1(5.3) | 15(12.1) | 13 (14.4) | 19 (26) | 11 (9) |
|  |  | *Reaction* | 30 (12.3) | - | - | - | - | - | - | - | - |
|  |  | *Abscess* | 27 (11.1) | - | - | - | - | - | - | - | - |
|  |  | *Pruritis* | - | - | - | - | 2 (10.5) | - | - | 4 (5) | - |
|  |  | *Bruising* | - | - | - | - | 4(21.1) | - | - | 6 (8) | - |
|  |  | *Haemorrhage* | - | - | - | 1(5.3) | 2(10.5) | - | - | 6 (8) | 1 (1) |
|  |  | *Haematoma* | - | 39(30) | - | - | 3(15.8) | 30(24.2) | 24 (26.7) | - | 39 (30) |
|  | **Hallucination** | | 42 (17.2) | - | - | - | - | - | - | 5 (7) | - |
|  | **Fall** | | 41 (16.8) | - | - | 9(7) | - | 8(6.5) | 12 (13.3) | 6 (8) | 9 (7) |
|  | **Anxiety** | | 29 (11.9) | - | - | - | - | 8(6.5) | 2 (2.2) | - | 6 (5) |
|  | **Depression** | | - | - | - | 2(10.5) | - | 8(6.5) | 1 (1.1) | - | - |
|  | **Dizziness** | | 25 (10.2) | - | - | - | - | - | - | - | - |
|  | **On/off** | | - | - | - | 5(26.4) | - | - | - | 6 (8) | 5 (4) |
|  | **Dyskinesia** | | - | - | - | 3(15.8) | - | 8(6.5) | 8 (8.9) | 8 (11) | 3 (2) |
|  | **Headache** | | - | - | - | 2(10.5) | 2(10.5) | - | - | - | 2 (2) |
|  | **Worsening PD** | | - | - | - | 2(10.5) | 1(5.3) | - | - | - | - |
|  | **Orthostatic hypotension** | | - | - | - | - | 2(10.5) | - | - | - | - |
|  | **Urinary tract infection** | | - | - | - | - | - | 13(10.5) | 7 (7.8) | - | - |
|  | **Nausea** | | - | - | - | - | - | 15(12.1) | 3 (3.3) | - | - |
|  | **Fatigue** | | - | - | - | - | - | 7(5.6) | - | - | - |
|  | **Contusion** | | - | - | - | - | - | 3(2.4) | 5 (5.6) | - | - |
|  | **Back pain** | | - | - | - | - | - | 3(2.4) | 5 (5.6) | - | - |
|  | **Balance disorder** | | - | - | - | - | - | - | - | 4 (5) | - |
|  | **Constipation** | | - | - | - | - | - | - | - | 4 (5) | - |
|  | **Peripheral swelling** | | - | - | - | - | - | - | - | 4 (5) | - |

**Table S3: Adherence and withdrawals data from each study. NB in Aldred 2023 there is inconsistency between the N values used here (N=137 completed treatment) and those used in our synthesis (N=116 for whom data were available). This reflects the different values used in the original paper, as outcome data was not available for all participants that completed treatment. This missing data has been considered in our risk of bias assessment for the study.**

| **Study** | **Completion rate (%)** | **Discontinuation due to withdrawal of consent (%)** | **Discontinuation due to lack of efficacy (%)** | **Discontinuation due to adverse events (%)** |
| --- | --- | --- | --- | --- |
| **Aldred et al (2023)^21^** | 56.1 | 12.3 | 4.5 | 23 |
| **Espay et al (2024)^45^** | 94 | 0.8 | 0 | 5.5 |
| **Giladi et al (2021)^30-32^** | 100 | NA | NA | NA |
| **LeWitt et al (2022)^29^** | 100 | NA | NA | NA |
| **Olanow et al (2021)^34-37^** | 87 | 2.6 | 5.2 | 5.2 |
| **Poewe et al (2021)^33^** | 56.1 | 19.6 | Not reported. | 17.3 |
| **Soileau et al (2022)^22-28^** | 78 | Not reported. | Not reported. | Not reported. (22 patients withdrew with an AE) |

**Appendix S1- Search Strategy**

We will search the following electronic databases:

- MEDLINE Ovid (from 1946)
- Embase Ovid (from 1974)
- Cochrane Central Register of Controlled Trials (CENTRAL)
- International Clinical Trials Registry Platform (ICTRP)
- Clinicaltrials.gov

Search date: 11/09/23

We will search databases with strategies we developed in consultation with an information specialist. Strategies for all databases are modelled on the MEDLINE search illustrated below:

| 1 | (levodopa or L-dopa or "L dopa" or 3-Hydroxy-L-tyrosine or "3 Hydroxy L tyrosine" or L-3,4-Dihydroxyphenylalanine or "L 3,4 Dihydroxyphenylalanine" or levopa or larodopa or dopaflex or dopar or foscarbidopa or foslevodopa or carbidopa or infudopa).ti,ab,kf. |
| --- | --- |
| 2 | *levodopa/ or *carbidopa plus levodopa/ |
| 3 | 1 or 2 |
| 4 | subcutaneous drug administration/ |
| 5 | subcutaneous*.ti,ab,kf. |
| 6 | 4 or 5 |
| 7 | 3 and 6 |
| 8 | Parkinson disease/ |
| 9 | (parkinson* or pd).ti,ab,kf. |
| 10 | 8 or 9 |
| 11 | 7 and 10 |
| 12 | (rat or rats or mouse or mice or swine or porcine or murine or sheep or lambs or pigs or piglets or rabbit or rabbits or cat or cats or dog or dogs or cattle or bovine or monkey or monkeys or trout or marmoset$1).ti. and animal experiment/ |
| 13 | Animal experiment/ not (human experiment/ or human/) |
| 14 | 12 or 13 |
| 15 | 11 not 14 |
| 16 | conference*.pt. or conference abstract/ |
| 17 | 15 not 16 |
